# Dynamic optimal experimental design yields marginal improvement over steady‐state results for computational maximisation of regulatory T‐cell induction in ex vivo culture

**DOI:** 10.1049/iet-syb.2018.5014

**Published:** 2018-12-01

**Authors:** Andrew Sinkoe, Arul Jayaraman, Juergen Hahn

**Affiliations:** ^1^ Department of Biomedical Engineering Rensselaer Polytechnic Institute 110 8th Street Troy New York USA; ^2^ Center for Biotechnology and Interdisciplinary Studies, Rensselaer Polytechnic Institute 110 8th Street Troy New York USA; ^3^ McFerrin Department of Chemical Engineering Texas A&M University College Station Texas USA; ^4^ Department of Chemical and Biological Engineering Rensselaer Polytechnic Institute 110 8th Street Troy New York USA

**Keywords:** patient treatment, molecular biophysics, proteins, cellular biophysics, diseases, Tregs relative, optimal input function, dynamic cytokine profiles, optimal constant concentrations, IL‐23, computational maximisation, regulatory T‐cell induction, inflammatory bowel disease, viable therapeutic option, local microenvironment, Treg differentiation, single concentrations, predicted induction, dynamic optimal experimental design, interleukin‐2, IL‐6, transforming growth factor‐β

## Abstract

The isolation of T cells, followed by differentiation into Regulatory T cells (Tregs), and re‐transplantation into the body has been proposed as a therapeutic option for inflammatory bowel disease. A key requirement for making this a viable therapeutic option is the generation of a large population of Tregs. However, cytokines in the local microenvironment can impact the yield of Tregs during differentiation. As such, experimental design is an essential part of evaluating the importance of different cytokine concentrations for Treg differentiation. However, currently only single, constant concentrations of the cytokines have been investigated. This work addresses this point by performing experimental design in silico which seeks to maximize the predicted induction of Tregs relative to Th17 cells, by selecting an optimal input function for the concentrations of TGF‐β, IL‐2, IL‐6, and IL‐23. While this approach sounds promising, the results show that only marginal improvements in the concentration of Tregs can be achieved for dynamic cytokine profiles as compared to optimal constant concentrations. Since constant concentrations are easier to implement in experiments, it is recommended for this particular system to keep the concentrations constant where IL‐6 should be kept low and high concentrations of TGF‐β, IL‐2, and IL‐23 should be used.

## 1 Introduction

Chronic inflammation, and specifically immune‐mediated inflammatory diseases, affects 5–7% of people in Western society and can result in irreversible damage to tissue and organs [1–3]. One mechanism underlying some cases of chronic inflammation is an imbalance of different T cell populations, such as, e.g. effector T cells and regulatory T cells (Tregs), where a person's Tregs are insufficient in number to regulate the inflammatory response [4–8].

Cell‐based therapeutics has been proposed as a potential treatment for chronic inflammation [5, 9–13]. However, treatments that show long‐term positive effects are rare, and effective treatments do not exist for some conditions such as inflammatory bowel disease [4, 9, 10, 14–16]. One potential treatment strategy is to isolate a population of a patient's own naive T cells, induce the cells *ex vivo* to differentiate into a high percentage of Tregs, and subsequently re‐transfer the differentiated T cells back into the patient at the site(s) of chronic inflammation [13]. The rationale is that the Tregs would be present at a sufficient abundance to regulate and mitigate chronic inflammation.

An important consideration involved in this proposed treatment modality is the question of how to maximise the number of Tregs induced from naive T cells in *ex vivo* culture. Maximising Treg induction is especially important due to the fact that naive T cells can also differentiate into T‐helper‐17 (Th17) cells, which have been shown to increase inflammation rather than mitigate it [5, 6, 8]. As a result, it is possible to generate a mixed population containing different T‐cell subtypes (e.g. Tregs, Th1, Th2, and Th17) which reduces the yield of Tregs.

Previous studies have shown that the set of cytokines present in the local extracellular environment of naive T cells influences differentiation fate [4–6, 13, 17, 18]. In particular, evidence has suggested that transforming growth factor β (TGF‐β) in combination with interleukin‐6 (IL‐6) induces the Th17 phenotype, while TGF‐β without IL‐6 induces Tregs, with IL‐2 and IL‐23 also playing a potential role [5]. In order to generate sufficient quantities of Tregs, it is necessary to maximise production of Tregs *in vitro* or *ex vivo* by exposing the T cells to an optimal concentration of different cytokines. These cytokines are typically used at a single, constant concentration throughout the *in vitro* differentiation phase [13]. However, since the different T‐cell subtypes also produce a wide array of cytokines, it is likely that the cytokine concentration in the extracellular milieu is not constant. Thus, a large space of experimental conditions is often used to identify optimal T‐cell differentiation conditions. As this requires a repeated evaluation of different experimental conditions, many designs involve an *in silico* component [5]. Since dynamic input profiles have been shown to significantly outperform steady‐state input profiles for some systems, e.g. certain biochemical reactions [19], the focus of this work is on *in silico* model‐based experimental design to determine optimal experiments that can take time‐varying cytokine input profiles into account for the particular system under investigation.

In order to perform this investigation, the mathematical model by Carbo *et al.* [5] is used. This model captures and quantifies the experimental observations that have led to characterisation of naive T‐cell differentiation fate based on extracellular cytokine stimulations. This model consists of a system of ordinary differential equations (ODEs) representing the signal transduction dynamics of naive T cells responding to extracellular cytokine signalling [5]. The model contains 59 ODEs corresponding to concentrations of the molecular species involved in signal transduction for differentiation of naive T cells [5]. Solving the ODEs numerically for different extracellular cytokine compositions allows for plotting the dynamic trajectories of any of the 59 signalling species. Three downstream species in the signalling pathway have been utilised extensively as biomarkers for Tregs and Th17 cells: (i) Forkhead box P3 (FOXP3) is a transcription factor used as a biomarker for Tregs; (ii) IL‐17, a cytokine, and (iii) RAR‐related orphan receptor *γ* isoform t (RORγt), a transcription factor, are both used as biomarkers for Th17 [4, 5, 8, 20].

**Fig. 1:**
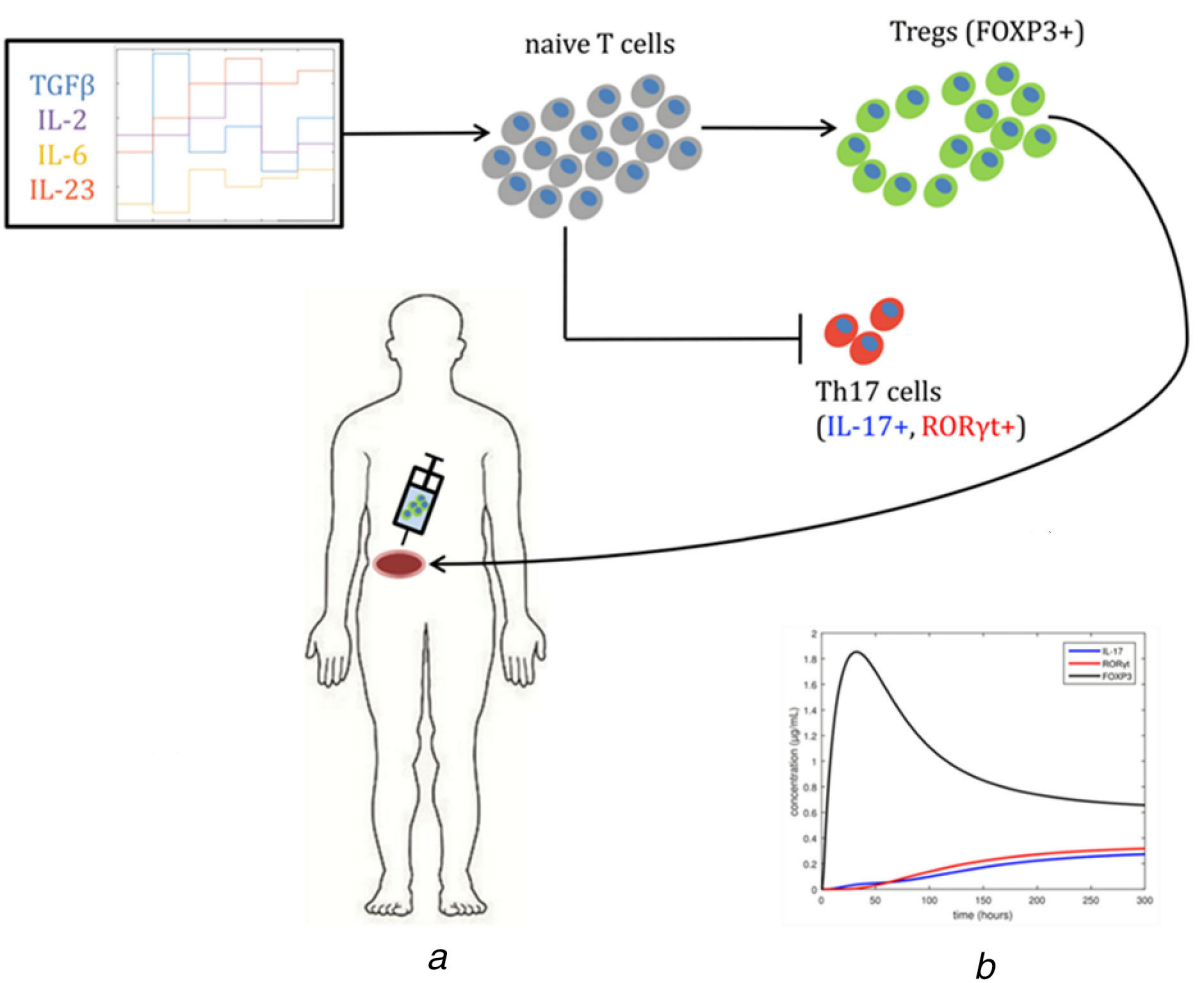
Illustration of the optimal experimental design problem to maximise Treg induction and simultaneously minimise Th17 induction from naive T cells in ex vivo culture ***(a)*** Schematic of differentiation and transplantation process, ***(b)*** Plot of biomarkers for Treg and Th17 which represent the two cell types in the model

Using this model, an optimisation problem is formulated that seeks to maximise differentiation into Tregs *in silico* by determining optimal time‐dependent input functions for the cytokines TGF‐β, IL‐2, IL‐6, and IL‐23. The results of the optimal experimental design may be used for informing clinical *ex vivo* differentiation experiments in which the goal is to maximise Treg induction relative to Th17 induction for T‐cell re‐transplantation and treatment of chronic inflammation, as illustrated in Fig. 1. Furthermore, a comparison between the optimal time‐dependent and steady‐state input profiles is also made as time‐dependent profiles are significantly more challenging to implement experimentally and as such dynamic input profiles should only be used if significantly higher Treg concentrations can be achieved due to the dynamics.

This paper is structured as follows: Section 2 describes the optimal experimental design problem that is solved for maximising Treg induction relative to Th17 induction, Section 3 presents the results of optimal experimental design including a sensitivity analysis on the objective function of the optimisation problem; Sections 4 and 5 discuss implications and conclusions drawn from the results of optimal experimental design for this problem.

## 2 Formulation of the optimal experimental design problem

Optimal experimental design has been used extensively to maximise experimental efficiency by optimising experimental conditions [21–26]. The development and implementation of optimal experimental design methods can lead to experiments that provide maximal information gain with minimal resource usage [21–26]. However, the use of optimal experimental design in biological systems is a more recent development and is not as wide‐spread as in other fields [26]. Here, optimal experimental design is applied to the problem of maximising Treg induction from naive T cells and simultaneously minimising induction of Th17 in the same *ex vivo* population (see Fig. 1).

### 2.1 Model of T‐cell differentiation signalling

Carbo *et al.* [5] constructed a mathematical model of the signalling pathways controlling naive T‐cell differentiation. This model contains 59 ODEs of the form in (1), where ***x*** is the vector of state variables representing concentrations of signalling species in the model at time *t*, and ***u*** is the vector of inputs (see [5], supplementary information). The model also contains fitted parameters and initial conditions and can be downloaded from the BioModels database [27, 28]

(1) $$\frac{dx}{dt}=f\left(t,x,u\right)$$

The model was constructed based on experimental observations from the literature and was shown by Carbo *et al.* [5] to accurately predict qualitatively differentiated phenotype when naive T cells were induced with various combinations of extracellular cytokines for differentiation purposes. The differentiated T‐cell phenotypes include Tregs and Th17 cells. A representative example of the ODEs found in the system of model equations is given below for the rate of change of TGF‐β

(2) $$\begin{array}{rl} \frac{dx_{\text{TGF}\beta }}{dt} & =v_{\text{TGF}\beta }\left(\frac{u_{\text{TGF}\beta }^{2}}{u_{\text{TGF}\beta }^{2}+x_{\text{TGF}\beta }^{2}+0.001}-k_{\text{TGF}\beta }x_{\text{TGF}\beta }\right)\\ & \;-k_{1}x_{\text{TGF}\beta }x_{\text{TGF}\beta R}-k_{2}x_{\text{TGF}\beta \cdot \text{TGF}\beta R}+k_{1}x_{\text{TGF}\beta } \end{array}$$

Here, the subscripts refer to chemical species involved in the reactions to produce or consume TGF‐β, including TGF‐β itself and its receptor, and the parameters are defined in the supplementary information for [5]. The equations for the model were derived from mass action kinetics and modifications of Michaelis–Menten kinetics.

**Fig. 2:**
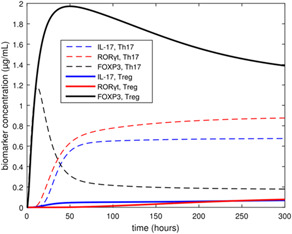
Biomarker trajectories for the Treg and Th17 biomarkers when input cytokine profile is Treg‐inducing (solid) or Th17‐inducing (dashed)

The model system of ODEs can be solved, using a numerical ODE solver, such as those contained in software packages such as MATLAB, to obtain predictions for time‐dependent trajectories *x* of the signalling molecules in the pathway if the input concentrations, ***u***, of extracellular cytokines is specified. The biomarkers FOXP3, IL‐17, and RORγt are used to represent Treg and Th17 concentrations in the *ex vivo* population (see Fig. 1). Fig. 2 shows plots of the concentrations of these biomarkers during a simulated *ex vivo* differentiation experiment for two different input cytokine compositions. The high FOXP3 expression and low IL‐17 and RORγt expression in Fig. 2 are indicative of the Treg phenotype, while the low FOXP3 expression and high IL‐17 and RORγt expression in Fig. 2 are indicative of the Th17 phenotype. Note that the biomarker concentrations are of the same order of magnitude, although they are not explicitly normalised in the model. The full model system of ODEs, as well as list of calibrated model parameter values, can be found in the supplementary information section of Carbo *et al.* [5].

### 2.2 Piecewise constant cytokine input functions

**Fig. 3:**
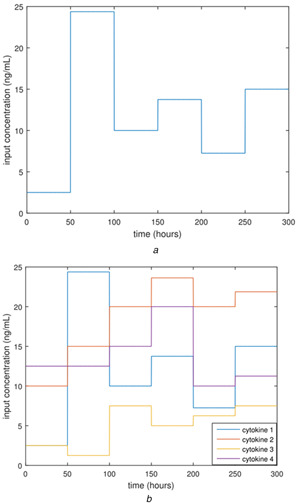
Generic piecewise constant input functions with one or multiple input cytokines ***(a)*** One input cytokine. The ***c*** matrix for this input function would be the 1 × 6 matrix [2.5, 24.4, 10, 13.75, 7.25, 15] ng/ml, ***(b)*** Four input cytokines. The ***c*** matrix for this vector input function would have the rows [2.5, 24.4, 10, 13.75, 7.25, 15] (cytokine 1), [10, 15, 20, 23.625, 20, 21.875] (cytokine 2), [2.5, 1.25, 7.5, 5, 6.25,7.5] (cytokine 3), [12.5, 12.5, 15, 20, 10, 11.25] (cytokine 4) ng/ml

In the original model by Carbo *et al.*, the cytokine input concentrations are constants, set at values that determine the concentration trajectories of the signalling molecules in the pathway. In this study, time‐dependent input functions are used instead of the constant input functions for four of the cytokines. The underlying hypothesis is that by optimising time‐dependent input functions, the *ex vivo* induction of Tregs from naive T cells will be increased relative to induction of Th17 compared to the case where only constant inputs are used. To test this hypothesis *in silico*, piecewise constant input functions were introduced into the model for the cytokines TGF‐β, IL‐2, IL‐6, and IL‐23, replacing the constant input functions for these cytokines. Piecewise constant functions were chosen because they are time dependent but can be tractably implemented in *ex vivo* culture experiments. The form of piecewise constant functions is shown in (3) below, where the function is a vector with each element corresponding to a cytokine. An example of a piecewise constant input function with one input or multiple inputs is shown in Figs. 3 *a* and *b*, respectively.

(3) $$\begin{array}{rl} u_{i}\left(t\right) & =\sum_{j=1}^{r}c_{i,j}\text{step}\left(t-\left(j-1\right)\Delta t\right)-\sum_{j=1}^{r}c_{i,j}\text{step}\left(t-j\Delta t\right)\\ & =c_{i,1}\text{step}\left(t\right)+\sum_{j=2}^{r}\left[c_{i,j}\text{step}\left(t-\left(j-1\right)\Delta t\right)\right.\\ & \;\left.-c_{i,j-1}\text{step}\left(t-\left(j-1\right)\Delta t\right)\right]-c_{i,r}\text{step}\left(t-r\Delta t\right)\\ & =c_{i,1}\text{step}\left(t\right)+\sum_{j=2}^{r}(c_{i,j}-c_{i,j-1})\text{step}\left(t-\left(j-1\right)\Delta t\right)\\ & \;-c_{i,r}\text{step}\left(t-r\Delta t\right) \end{array}$$

In (3), $c_{i,j}$ is the (i,j) element of the matrix ***c*** which contains the input concentration levels of the cytokines in each of the *r* time intervals. The index *i* represents the cytokine TGF‐β, IL‐2, IL‐6, or IL‐23; *j* represents the time interval; ‘step’ represents the Heaviside step function; $u_{i}$ is the input concentration level of cytokine *i* at time *t*; and Δt is the duration of the time intervals for each concentration level. For the present problem, a number of values for *r* were investigated; however, only the results for *r*  = 6 are discussed in detail here as they are representative for this investigation. As the experimental duration is 300 h, this results in Δ*t*  = 300 h/6 = 50 h, and ***c*** is a 4 × 6 matrix of concentration levels for the four cytokines over the six time intervals of the *ex vivo* differentiation culture (see Fig. 3).

The rationale for introducing time‐dependent input functions is that biological signalling systems are inherently dynamic and may, therefore, respond differently to time‐varying extracellular inputs.

### 2.3 Objective function for maximisation of Treg induction

The mathematical expression of the objective function is given as

(4) $$\text{obj}\left(c\right)={\left[\text{FOXP}3\right]}_{\text{final}}-{\left[\text{IL}-17\right]}_{\text{final}}-{\left[\text{ROR}\gamma t\right]}_{\text{final}},$$

where ***c*** is the matrix of optimisation variables defined in (3). Here, each term is the final concentration of a biomarker in the *ex vivo* differentiation culture. The final concentration is chosen because it is at the final time point that the Tregs in culture are harvested for re‐transplantation into the patient. Note that the objective function is the difference between Treg biomarkers (FOXP3) and Th17 biomarkers (IL‐17 and RORγt), which represents the relative difference between Tregs and Th17 cells differentiated in culture. Note further that Th17 differentiation is represented by the sum of both its biomarkers, IL‐17 and RORγt. This objective function is maximised in the optimisation problem to determine predicted optimal experimental conditions for obtaining the maximal number of differentiated Tregs relative to differentiated Th17 cells in the *ex vivo* culture. Finally, it should be noted that other objective functions representing relative Treg induction to Th17 induction are possible. However, the objective function in (4) was chosen so that $\text{obj}\left(c\right)$ is linear in the final biomarker concentrations, in contrast to a different objective function which would be non‐linear in these concentrations, e.g. ratios of the biomarker concentration. Using a linear objective function facilitates the derivation of the objective function gradient, described below. As one goal of this paper was to implement an optimisation solution using a method for calculating the analytical gradient, the objective function was chosen as (4), above.

Bounds were placed on the concentrations of the input cytokines, specifying a range of allowable input concentrations. The input concentrations were bounded between 0 and 25 ng/ml for the optimisation problem in order to avoid selecting concentration levels that are too high and therefore possibly cytotoxic to the naive T cells. The above formulation results in the optimisation problem

(5) $$\begin{array}{cc} \text{maximise}\;\; & \text{obj}\left(c\right)\;\;\;\;\;\\ \text{subject}\;\text{to}: & \text{model}\;\text{equations};\;\\ \;\;\;\; & 0\leq c\leq 25\;\text{ng}/\text{mL}. \end{array}$$

It should be noted that a different number of time intervals, *r*, and thereby different sampling times Δt, as well as different bounds on the inputs, have also been investigated. However, only the most meaningful results, in terms of biological significance and for representation purposes, are discussed in this work.

### 2.4 Gradient of the objective function for maximisation of Treg induction

The gradient of (4) is written as

(6) $$g\left(c\right)=\frac{\partial {\left[\text{FOXP}3\right]}_{\text{final}}}{\partial c}-\frac{\partial {\left[\text{IL}-17\right]}_{\text{final}}}{\partial c}-\frac{\partial {\left[\text{ROR}\gamma t\right]}_{\text{final}}}{\partial c}.$$

Since the final concentrations of the biomarkers are not algebraic functions of ***c***, and can only be computed by numerically solving the system of model ODEs, the derivatives in the gradient (6) cannot be computed by directly applying differentiation rules, a similar scenario to that presented in [29]. To calculate these derivatives, a method involving sensitivity analysis, described below, was implemented. This was done because the three terms in the gradient (6) are sensitivity coefficients which can be computed from the model.

Sensitivity analysis, and in particular, local sensitivity analysis, is the calculation of the differential effect of model parameters on model output quantities [26, 30]. For the present model, the sensitivity coefficients for differential changes in signalling species concentration with respect to input function concentration levels can be considered. These sensitivity coefficients take the form

(7) $$\frac{\partial x_{k}\left(t\right)}{\partial c},$$

where $x_{k}$ is the concentration of signalling species *k*. To compute the sensitivity coefficients as a function of time for every state variable $x_{k}$ in the model, the sensitivity equations (8) can be solved simultaneously with the model ODEs (1) using a fixed‐step ODE solver, such as the MATLAB function ode5, a fixed‐step fifth‐order Runge–Kutta solver.

(8) $$\frac{d}{dt}\frac{\partial x}{\partial c}=\frac{\partial f}{\partial x^{T}}\frac{\partial x}{\partial c}+\frac{\partial f}{\partial c}$$

In (8), ***x*** is the vector of state variables and ***f*** is the vector of slope functions on the right side of the ODE model (1).

When the sensitivity equations are solved for a given input matrix ***c***, the sensitivity coefficients that comprise the terms of the gradient (6) will have been evaluated for that given ***c***, and the gradient can, therefore, be evaluated by selecting only those sensitivity coefficients from the larger array containing all of the sensitivity coefficients of the state variables at every time point in the ODE solution. This procedure was performed in MATLAB to evaluate the gradient for every iteration of the optimisation problem.

To evaluate the gradient, it is necessary to evaluate the individual terms of (8), namely $\partial f/\partial x^{T}$ and ∂f/∂c. The first of these is the Jacobian of the model ODE system, which is computed by taking the individual partial derivatives of each slope function $f_{k}$ with respect to each state variable $x_{l}$. The second term ∂f/∂c is calculated as

(9) $$\begin{array}{rl} \frac{\partial f_{k}}{\partial c_{i,j}} & ={\left.\frac{\partial f_{k}}{\partial u_{i}}\right|}_{t}{\left.\frac{\partial u_{i}}{\partial c_{i,j}}\right|}_{t}\\ & ={\left.\frac{\partial f_{k}}{\partial u_{i}}\right|}_{t}\left[\text{step}\left(t-\left(j-1\right)\Delta t\right)-\text{step}\left(t-j\Delta t\right)\right] \end{array}$$

(10) $${\left.\frac{\partial f_{k}}{\partial u_{i}}\right|}_{t}=\left\{\begin{array}{cc} \frac{2v_{i}u_{i}}{u_{i}^{2}+x_{k}^{2}+0.001}*\left(1-\frac{u_{i}^{2}}{u_{i}^{2}+x_{k}^{2}+0.001}\right), & k=i\in \left\{\text{TGF}\beta ,\text{IL}2,\text{IL}6,\text{IL}23\right\}\\ 0\;\;\;\;\;\;\;\;\;\;\;\;\;\;\;\; & \text{otherwise}\;\;\;\;\;\;\;\; \end{array}\right.,$$

where (10) is due to the consistent form of the model equations which causes the partial derivative to be of the same form for each *i*, and $v_{i}$ is a constant parameter in the model, specific to each cytokine *i*. One can observe how (10) is obtained by taking the derivative of (2) when i=k=TGFβ. Note that the value of 0.001 in the denominators of (10) was determined by Carbo *et al.* [5] to calibrate the model with experimental data.

## 3 Solution of the optimal experimental design problem

A non‐linear programming problem was formulated using the objective function (4) and gradient (6). This problem was set up in MATLAB and the optimisation problem was solved using IPOPT, a state‐of‐the‐art interior point algorithm [31]. By supplying the exact gradient to IPOPT rather than requiring the solver to approximate the gradient with finite differences, the solution speed was increased by an order of magnitude: whereas IPOPT found the optimal solution in 180 s when the exact gradient was available, it did not determine a solution before exceeding the maximum time limit of 2000 s when the gradient was approximated. This was due to the fact that finite difference approximations for partial derivatives in the gradient require the model ODE system to be solved multiple times per iteration. Furthermore, a larger number of iterations were required for the solution of the optimisation problem using numerical gradients because the finite difference approximations cause the solver to move in directions based on a less accurate gradient calculation. Lastly, a particle swarm optimisation routine from the OPTI toolbox for MATLAB (https://www.inverseproblem.co.nz/OPTI/) was also used for solving this problem to verify that the found results are not just local solutions or specific to the particular solver used. As particle swarm optimisation found the same results returned by IPOPT only one set of solutions is shown in this work.

**Fig. 4:**
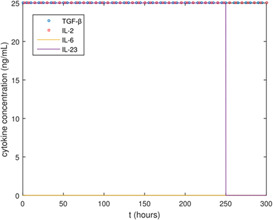
Optimal cytokine input functions

The optimal solution is plotted in Fig. 4, showing the optimal input cytokine concentration functions. The optimal TGF‐β input function is constant at the upper concentration bound, indicating that a high concentration is optimal for TGF‐β; this is also true for IL‐2. The optimal concentration for IL‐6 is very low, approximately zero, as expected from previous investigations [5, 6]. IL‐23, however, remains at high values for the first five time intervals and is then decreased to approximately zero for the final time interval.

**Fig. 5:**
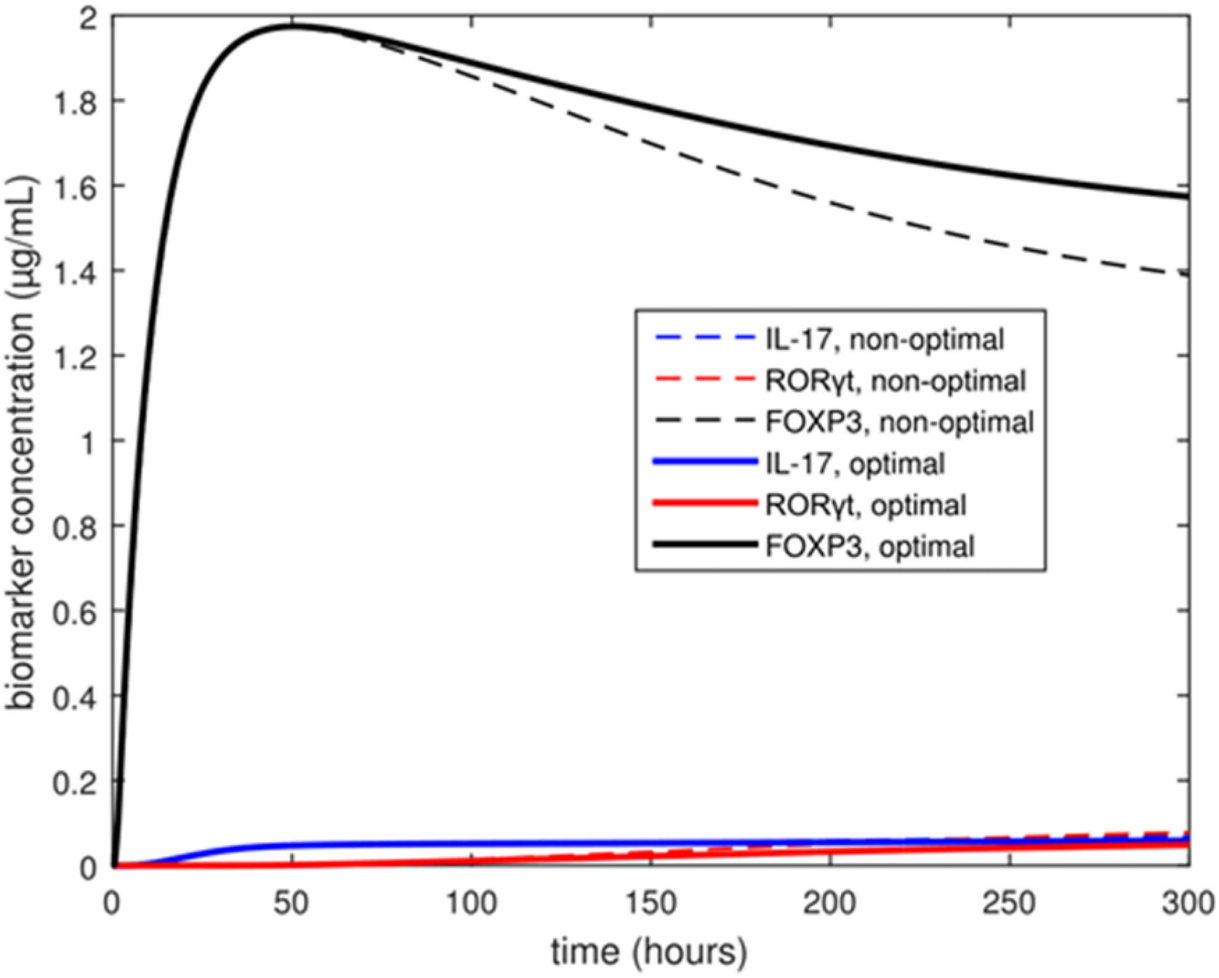
Biomarker trajectories for the Treg and Th17 biomarkers when input cytokine profile is optimal (solid) and, separately, constant (dashed)

A plot of the biomarkers in response to the optimal input function is shown in Fig. 5, superimposed on a plot of the biomarkers in response to a constant (non‐optimal) input profile. It can be seen from this plot that the optimal biomarker response only provides a marginal improvement from a non‐optimal biomarker response obtained from constant cytokine profiles. This suggests that constant cytokine profiles may suffice for an *ex vivo* differentiation experiment, as long as the cytokine inputs are at constant levels that are conducive to Treg induction, i.e. high TGF‐β and low IL‐6.

**Fig. 6:**
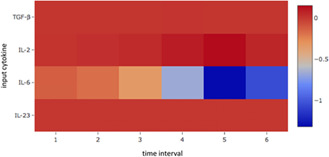
*Heatmap showing relative sensitivity coefficients of objective function with respect to input cytokine concentration levels. The absolute sensitivity coefficients were normalised by* (1) *the values in the matrix **c** which were all 12.5 ng/ml and* (2) *the value of the objective function when evaluated at the same value of the **c** matrix*

To test the importance of each of the four cytokines, a sensitivity analysis was carried out to measure the effect of each cytokine concentration level on the objective function. Since the gradient of the optimisation problem contains the sensitivity coefficients, this sensitivity analysis was performed by evaluating the gradient of the objective function for a value of ***c*** within the range of allowable concentration levels. The value of ***c*** was chosen as 12.5 ng/ml for each element of ***c***, half of the upper bound concentration. Results of the sensitivity analysis are shown in Fig. 6. The heatmap shows that IL‐6 is the only cytokine that has a substantial effect on the objective function among the four cytokines considered. This finding is consistent with the previously established experimental and computational evidence that IL‐6 is the cytokine that has the strongest influence on the outcome, i.e. Treg or Th17, in the presence of TGF‐β.

## 4 Discussion

The optimal experimental design using dynamic inputs led to marginally improved concentrations of Treg biomarkers compared to constant inputs for the four cytokines TGF‐β, IL‐2, IL‐6, and IL‐23. These cytokines were selected due to their known role in naive T‐cell differentiation into Tregs and Th17 cells. It was hypothesised that introducing time‐dependent input functions for these cytokines would improve Treg induction relative to Th17 induction, compared to constant input functions. The small improvement in the Treg maximisation objective function appears to be achieved by maintaining high TGF‐β and IL‐2 concentration, low IL‐6 concentration, and by changing the IL‐23 concentration from high to low before the final time interval. The comparison between optimal biomarker trajectories and non‐optimal biomarker trajectories showing that the objective was only improved marginally by the optimal input function profile suggests that nearly optimal Treg induction relative to Th17 induction can be achieved using constant cytokine input profiles. As constant input profiles are easier to use experimentally, these results indicate that the benefit from using dynamic input profiles, at least for this particular system, might not be worth the additional experimental efforts compared to constant inputs. However, it is important to note that the results are dependent upon the model used and that an experimental investigation to validate these *in silico* findings should be done.

Previous observations [5, 6] indicate that IL‐6 has the strongest influence on differentiation into Treg and Th17 in the presence of TGF‐β. The sensitivity analysis results (Fig. 6) support this observation, showing that the objective function is substantially more sensitive to IL‐6 input concentration changes than to changes in the other cytokines considered. This suggests that it is important to keep extracellular IL‐6 concentration low throughout an *ex vivo* Treg induction experiment, especially during the later time points, at which the sensitivity coefficients have a larger magnitude (Fig. 6).

## 5 Conclusion

This paper investigated the maximisation of regulatory T‐cell induction using experimental design applied to a model from the literature. Optimal piecewise constant cytokine input functions led to increased Treg induction *in silico* compared to constant input concentrations. However, constant inputs may suffice experimentally, as the predicted improvement in Treg induction from using piecewise constant inputs was only marginal compared to constant inputs.

IL‐6 concentration was found to be the most important factor in producing a large fraction of Tregs, and the results are consistent with existing literature, suggesting that the concentration of this cytokine should be kept as low as possible. The *in silico* results suggest that TGF‐β and IL‐2 should be kept at high concentrations while varying IL‐23 from high to low in the last time interval of the experiment may maximise Treg induction relative to Th17 induction. Due to the *in silico* nature of this work, these results will need to be validated experimentally.
